# The Hospital World

**Published:** 1910-06-18

**Authors:** 


### The Hospital World.

### Elections and Appointments.

Prince Arthur of Connaught has been appointed a vice-president of the Middlesex Hospital.

Dr. Burnet has been appointed medical superintendent of the Aitken Sanatorium, Bury.

Mr. Councillor Stockdale has been appointed a member of the Bury and District Joint Hospital Board.

Mr. R. W. M'Dowell and Mrs. M'Dowell have qualified as life governors of the Royal Victoria Hospital, Belfast.

Mrs. Ainley Walker has been elected a member of the General and House Committees of the Oxford Eye Hospital.

Mrs. Love, of Hawkhills, has been elected lady president of the St. Monica's Cottage Hospital, Easingfold, Yorkshire.

Mr. Fergus, Mr. McCrindle, and Mr. Young have been re-elected governors of the Incorporated Glasgow Dental Hospital.

Mr. Savage French has been re-elected chairman of the Queenstown General Hospital; Mr. R. Swanton, J.P., has been elected hon. treasurer and Dr. Ring hon. secretary.

Lord Langford, K.C.V.O., has been re-elected chairman of the Management Committee of the Adelaide Hospital, Dublin, and F. T. Heuston, Esq., M.D., vice-chairman, for the next twelve months.

Judge Orr, Mr. J. Redmond-Blood, Mr. Benjamin F. Eustace, J.P., the Rev. Fergus Greer, M.A., and Mr. W. J. Arnold have been elected to the committee of the Drumcondra Hospital, Whitworth Road, Dublin.

Mr. C. E. Bosquet has been elected president of a new scheme known as the Hospital Weekly Contribution Committee for the benefit of the Warneford Hospital and Convalescent Homes of Leamington. Mr. J. Stiles has been chosen hon. secretary.

At a special meeting of the Board of Governors of Cheltenham General Hospital, held on June 14, a vacancy amongst the honorary physicians, consequent on the resignation of Dr. R. Kirkland, was filled by the appointment of Dr. J. R. Collins. The successful candidate is a M.A., M.D., B.Ch. of Dublin University, and was formerly a Resident Medical Officer at Sir Patrick Dun's Hospital in Dublin, Resident Surgeon at the Cheltenham General Hospital in 1902, Surgeon in Charge of the Branch Dispensary in 1903 and 1904, and in 1909 was appointed Honorary Clinical Pathologist to the Hospital. At the same meeting Dr. E. J. Tatham, of Beaufort House, Cheltenham, was elected Honorary Anæsthetist to the Institution. Dr. Tatham, who in the latter part of the eighties held the post of Resident Surgeon to the Hospital, is a M.D., B.C. of Cambridge University, and M.R.C.S.Eng.

### Personalia.

In the death of Mr. Edward Eastwood, at the age of 84, the Chesterfield and North Derbyshire Hospital loses a benefactor who has done many good services to it in his time. Apart from his steady if unobtrusive general support, the new medical wing, which was built in 1902, was a present from him. He gave also the site for a nurses' home. Though it is only his work for the hospital which we can touch on here, his benefactions covered human activities of all sorts, and most people in Derbyshire have good cause to remember him. Certainly his name will not be forgotten at the Chesterfield and North Derbyshire Hospital.

There were some 116 candidates for the vacant post of Secretary to the Sussex County Hospital, Brighton. Of these three were selected to appear before the Elective Committee on Wednesday, June 8. The selected candidates were: Mr. J. Stephen Neil, Secretary Wolverhampton and Staffordshire General Hospital, late of the Portsmouth Hospital and the Great Northern Central Hospital; Mr. Reginal B. Jay, Assistant-Secretary Sussex County Hospital, Brighton, late of the Norfolk and Norwich Hospital; Mr. E. C. Smith, F.C.I.S., Secretary North Ormesby Hospital, Middlesbrough, late of Swansea General and Eye Hospital. After interviewing the three candidates Mr. Jay was appointed Secretary.

Mr. A. E. Thomas has been appointed Secretary to the Hampstead General Hospital, with which is amalgamated the North-West London Hospital. Here is something of his record. After some years of business training in a London office Mr. Thomas first took up hospital work in April 1900 at Gravesend, and held the post of Secretary to the General Hospital there with great success for eight years. In May 1908, with the idea of obtaining wider experience, and in order to gain a more intimate knowledge of London methods of hospital administration, Mr. Thomas obtained the Assistant Secretaryship of the Royal Free Hospital, where his excellent work has been very highly appreciated both by the board and Mr. Conrad Thies, who much regret to lose his services.

The new Secretary of the Belgrave Hospital for Children, Mr. T. W. Gregg, may be considered a lucky, as he is certainly a capable, man. So far his life has been a continual promotion. Ten years ago, as a boy almost, he became an institutional worker, entering the East London Hospital for Children, Shadwell, which has had the training of him. First as junior clerk, when Mr. Thomas Hayes was Secretary, he learnt the rudiments of a secretary's work, and later under Mr. Wilcox was promoted to the senior clerkship. The result is that, at the age of twenty-six, Mr. Gregg finds himself secretary to a London institution with forty beds, treating a total number of over 12,000 patients in the course of a year. There is nothing so useful as an early beginning, and if many people are congratulating Mr. Gregg to-day on the quick reward his abilities have won for him, his previous record seems to prophesy that at the end of a year or two the Belgrave Hospital will be saying that the success of the appointment was not only on his side.

Mr. D. D. Kirkaldy has been appointed Secretary to the West End Hospital for Diseases of the Nervous System, to fill the place held till recently by Mr. Alfred Wise. Mr. Kirkaldy is a London man, and a London man in a very genuine sense. First as Honorary Secretary to the South Wimbledon, Merton, and District Cottage Hospital, to which he went, we understand, in 1904, then transferred in another capacity to the London Hospital, he gained experience in the duties of institutional work; and besides this he has the distinction of being a Bachelor of Arts of the London University. Thus a small hospital in the suburbs, the largest hospital in the city, and the University with perhaps the greatest academic prestige, have been the places of Mr. Kirkaldy's education. If this is not a varied enough training for the versatility required in a hospital secretary, it would be hard to know what else to suggest. Mr. Kirkaldy will find plenty of opportunity as Secretary of the Belgrave Hospital for putting it to the test, at any rate.

